# Enzymatic Epoxidation of Long-Chain Terminal Alkenes by Fungal Peroxygenases

**DOI:** 10.3390/antiox11030522

**Published:** 2022-03-08

**Authors:** Esteban D. Babot, Carmen Aranda, Jan Kiebist, Katrin Scheibner, René Ullrich, Martin Hofrichter, Angel T. Martínez, Ana Gutiérrez

**Affiliations:** 1 Instituto de Recursos Naturales y Agrobiología de Sevilla, Consejo Superior de Investigaciones Científicas (CSIC), 41012 Seville, Spain; edbabot@irnase.csic.es; 2 Johnson Matthey, Cambridge Science Park U260, Cambridge CB4 0FP, UK; carmen.aranda@matthey.com; 3 Institute of Biotechnology, Brandenburg University of Technology Cottbus-Senftenberg, 01968 Senftenberg, Germany; jan.kiebist@b-tu.de (J.K.); katrin.scheibner@b-tu.de (K.S.); 4 Unit of Bio- and Environmental Sciences, International Institute Zittau, TU Dresden, 02763 Zittau, Germany; ullrich@tu-dresden.de (R.U.); martin.hofrichter@tu-dresden.de (M.H.); 5 Centro de Investigaciones Biológicas “Margarita Salas”, Consejo Superior de Investigaciones Científicas (CSIC), 28040 Madrid, Spain; atmartinez@cib.csic.es

**Keywords:** peroxygenases, oxyfunctionalization, epoxidation, terminal alkenes, epoxides

## Abstract

Terminal alkenes are among the most attractive starting materials for the synthesis of epoxides, which are essential and versatile intermediate building blocks for the pharmaceutical, flavoring, and polymer industries. Previous research on alkene epoxidation has focused on the use of several oxidizing agents and/or different enzymes, including cytochrome P450 monooxygenases, as well as microbial whole-cell catalysts that have several drawbacks. Alternatively, we explored the ability of unspecific peroxygenases (UPOs) to selectively epoxidize terminal alkenes. UPOs are attractive biocatalysts because they are robust extracellular enzymes and only require H_2_O_2_ as cosubstrate. Here, we show how several UPOs, such as those from *Cyclocybe* (*Agrocybe*) *aegerita* (*Aae*UPO), *Marasmius rotula* (*Mro*UPO), *Coprinopsis cinerea* (r*Cci*UPO), *Humicola insolens* (r*Hin*UPO), and *Daldinia caldariorum* (r*Dca*UPO), are able to catalyze the epoxidation of long-chain terminal alkenes (from C_12:1_ to C_20:1_) after an initial optimization of several reaction parameters (cosolvent, cosubstrate, and pH). In addition to terminal epoxides, alkenols and other hydroxylated derivatives of the alkenes were formed. Although all UPOs were able to convert and epoxidize the alkenes, notable differences were observed between them, with r*Cci*UPO being responsible for the highest substrate turnover and *Mro*UPO being the most selective with respect to terminal epoxidation. The potential of peroxygenases for epoxidizing long-chain terminal alkenes represents an interesting and green alternative to the existing synthesis technologies.

## 1. Introduction

The importance of epoxides in the chemical industry is based on their high reactivity. There are innumerable reactions that epoxides can undergo with a variety of chemical compounds, which makes them valuable intermediates in organic synthesis. The epoxidation of alkenes provides oxirane compounds (epoxides) that are essential raw materials in the production of fine chemicals (such as surfactants, epoxy resins, perfumes, plasticizers, pharmaceuticals, agrochemicals, polymers, cosmetics, or paints) and are vital intermediate compounds in organic synthesis [1,2,3]. In this regard, terminal alkenes are among the most attractive starting materials for chemical synthesis, as they are readily available in large-scale industrial processes and can be obtained on a smaller scale through a number of efficient, catalytic, and highly selective processes [4]. Furthermore, olefin epoxidation plays an important role in the valorization of biomass and byproducts of fossil oil refineries, yielding essential and versatile intermediate building blocks for the pharmaceutical, flavoring, and polymer industries [5].

For several years, investigation has focused on the use of ecologically friendly chemical oxidizers, such as H_2_O_2_, dioxygen (O_2_), organic peracids (R-CO_3_H), and alkyl hydroperoxides (R-O_2_H), in alkenes oxidative reactions [6,7,8,9,10,11,12,13]. The advantage of H_2_O_2_ as an oxidizer is the formation of water (H_2_O) as the only byproduct, with the disadvantage of requiring the use of metallic catalysts [14,15,16]. Chemoenzymatic epoxidation using lipases and H_2_O_2_ has also been reported for alkene epoxidation [17]. Nevertheless, another alternative for the epoxidation of terminal alkenes is the use of enzymes as reported for nonheme monooxygenases, such as toluene, styrene and methane monooxygenases (MMO) [18,19,20], chloroperoxidase [21], and an engineered cytochrome P450 monooxygenase (P450) [22], which are often used as microbial whole-cell catalysts [23]. However, whole-cell biotransformation typically requires long incubation times and is limited by the toxicity of the reaction compounds for cells. Conversely, nonheme monooxygenases (except MMO) are specific enzymes and, in most cases, need cosubstrates as electron donors. P450s, in addition to requiring NADH, often need an auxiliary flavin-enzyme module and have low stability. Moreover, although successful selective epoxidation of short-chain terminal alkenes has been reported with some of these enzymes, they are not able to epoxidize alkenes with chain lengths longer than eight carbons [18,19,20].

The main purpose of this study was to achieve the epoxidation of long-chain terminal alkenes with enzymes, particularly with fungal peroxygenases, also named unspecific peroxygenases (UPOs, EC.1.11.2.1), while taking advantage of the new enzymes discovered in the last few years [24]. UPOs represent a relatively new and appealing type of biocatalysts for organic synthesis, which, unlike P450s, are extracellular enzymes that are more stable and only require H_2_O_2_ for activation, with formation of H_2_O as a byproduct. UPOs have been shown to catalyze a variety of interesting oxygenation reactions, hydroxylation and epoxidation included [25,26], with more than 300 substrates already reported [27] including aromatic and heterocyclic substrates [28,29]; aliphatic compounds such as fatty acids, alkanes, fatty alcohols [25,30,31,32,33,34,35,36,37,38], steroids [39,40], and secosteroids [41,42]; and other flavor and fragrance compounds such as isophorone, ionones, and damascones [43,44]. The first UPO was described in the basidiomycetous fungus *Cyclocybe* (*Agrocybe*) *aegerita* [45] and, since then, a handful of other UPO enzymes have been isolated from other rather different wild-type species of Basidiomycota and Ascomycota, such as *Coprinellus radians* [46], *Marasmius rotula* [47], and *Chaetomium globosum* [40], which is indicative of their widespread occurrence in the fungal kingdom. In addition to these homologous wild-type (i.e., nonrecombinant) enzymes, there are other UPOs, e.g., from *Coprinopsis cinerea* and *Humicola insolens*, which are only known as recombinant proteins heterologously expressed by Novozymes A/S (Bagsvaerd, Denmark) in the mold *Aspergillus oryzae* [48]. Recently, a new UPO from the ascomycete *Daldinia caldariorum* has become available from Novozymes after gene expression in *A. oryzae*, being also expressible in *Escherichia coli* as a soluble and active enzyme [37]. The scarce studies on the reaction of these enzymes with terminal alkenes only include epoxidation of short alkenes (with low conversion rates) with *A. aegerita* UPO (*Aae*UPO) [33] or report unsuccessful epoxidation of a long terminal alkene (C_14:1_) with two different UPOs [32]. UPOs [33] and P450s [49] are inactivated during the reaction with terminal alkenes due to heme alkylation. In the present work, we aimed to expand these results, first studying the optimal conditions of the reactions to obtain epoxides from long-chain terminal alkenes (C_12:1_ to C_20:1_) and then exploring the potential of different fungal peroxygenases in these reactions.

## 2. Materials and Methods

### 2.1. Enzymes

*Aae*UPO (isoform II of 46 kDa), the first fungal peroxygenase purified and described in 2004 [45], was prepared as a wild-type enzyme from liquid cultures of *A. aegerita* TM-A1 grown in a soybean-peptone medium. *M. rotula* peroxygenase (*Mro*UPO; 32 kDa as monomeric enzyme), another wild-type UPO, was isolated from cultures of the respective agaric fungus (DSM-25031) [47]. The UPO of *C. globosum* (*Cgl*UPO; 36 kDa) is the third wild-type peroxygenase prepared from the liquid cultures of this ascomycetous mold (DSM-62110) [40].

The recombinant enzymes from *C. cinerea* (r*Cci*UPO; 44 kDa), *H. insolens* (r*Hin*UPO), and *D. caldariorum* (r*Dca*UPO) were provided by Novozymes A/S. r*Cci*UPO corresponds to the protein model 7249 from the sequenced *C. cinerea* genome available at JGI (http://genome.jgi.doe.gov/Copci1, accessed on 1 February 2022) used in several studies [32,39,41]. The sequences of r*Hin*UPO and r*Dca*UPO are included in Novozymes patents [48,50] and the former has already been used in oxyfunctionalization reactions [40,51]. These recombinant UPOs were expressed in *A. oryzae* [52]. All UPO proteins were purified by fast protein liquid chromatography (FPLC) using a combination of size-exclusion and ion-exchange chromatography on different anion and cation exchangers. Purification was confirmed by sodium dodecyl sulfate-polyacrylamide gel electrophoresis (SDS-PAGE) and UV–visible spectroscopy following the characteristic heme maximum of around 420 nm (Soret band of resting-state heme-thiolate proteins). Enzyme concentration was estimated according to the characteristic UV–visible band of the reduced UPO complex with carbon monoxide [53].

### 2.2. Model Compounds

Terminal alkenes, namely, 1-dodecene (C_12:1_), 1-tridecene (C_13:1_), 1-tetradecene (C_14:1_), 1-pentadecene (C_15:1_), 1-hexadecene (C_16:1_), 1-heptadecene (C_17:1_), 1-octadecene (C_18:1_), 1-nonadecene (C_19:1_), and 1-eicosene (C_20:1_), were used as substrates of the above UPOs. Tetradecane-1,2-diol and 1,2-epoxytetradecane were used as standards in gas chromatography-mass spectrometry (GC-MS) analyses. All reagents were purchased from Sigma-Aldrich (Saint Louis, MI, USA).

### 2.3. Enzymatic Reactions

Reactions (500 µL volume) with different model compounds (1 mM) were performed at 30 °C in 50 mM phosphate buffer at pH 5.5 or 7.0. Different proportions of acetone or acetonitrile (0–60%) as a cosolvent were tested. The enzyme concentration used was in the range of 2–3 µM. The cosubstrate H_2_O_2_ or *tert*-butyl hydroperoxide (*t*BuOOH) was continuously added with a syringe pump at 5 µL/h (within 24 h) or 60 µL/h (within 2 h) to give a concentration in the reaction mixture of 0.5–3 mM. In control experiments, substrates were treated under the same conditions (including cosubstrate) but without an enzyme. Products from enzymatic reactions were extracted with ethyl acetate, which was evaporated under nitrogen (N_2_) and derivatized with *N*,*O*-*bis*(trimethylsilyl)trifluoroacetamide (Supelco; Bellefonte, PA, USA) to be analyzed by GC-MS.

### 2.4. GC-MS Analyses

The analyses were performed with a Shimadzu (Kyoto, Japan) GC-MS QP 2010 Ultra system, using a fused-silica DB-5HT capillary column (30 m × 0.25 mm, 0.1 μm) from J&W Scientific (Folsom, CA, USA). The oven was heated from 50 °C (1.5 min) to 90 °C (2 min) at 30 °C min^−1^, and then from 90 °C to 250 °C (15 min) at 8 °C min^−1^. The injection was performed at 250 °C and the transfer line was kept at 300 °C. Compounds were identified by comparison of their mass spectra and retention times with commercial standards and/or by comparison of their mass spectra with those present in the Wiley and NIST libraries.

## 3. Results and Discussion

The oxirane ring of epoxides has been termed the “lord of the chemical rings” due to reactivity and relevance in different organic syntheses of industrial importance [54]. In the present study, the selective epoxidation of long-chain terminal alkenes by several UPOs from the basidiomycetous fungi *A. aegerita*, *C. cinerea,* and *M. rotula*; and the ascomycetous species *C. globosum*, *H. insolens,* and *D. caldariorum* was investigated (using GC-MS analysis). The work started with an optimization of the reaction conditions (cosolvent- and peroxide-type included) using one particular alkene, 1-tetradecene (C_14:1_), and one UPO (*Cgl*UPO). Then, the study was extended to the above-mentioned UPOs and, finally, several alkenes (C_12:1_ to C_20:1_) were tested as substrates of these UPOs under the optimized reaction conditions as described below.

### 3.1. Optimization of Reaction Conditions (Cosolvent and Cosubstrate) for Conversion of 1-Tetradecene by CglUPO

**Figure 1:**
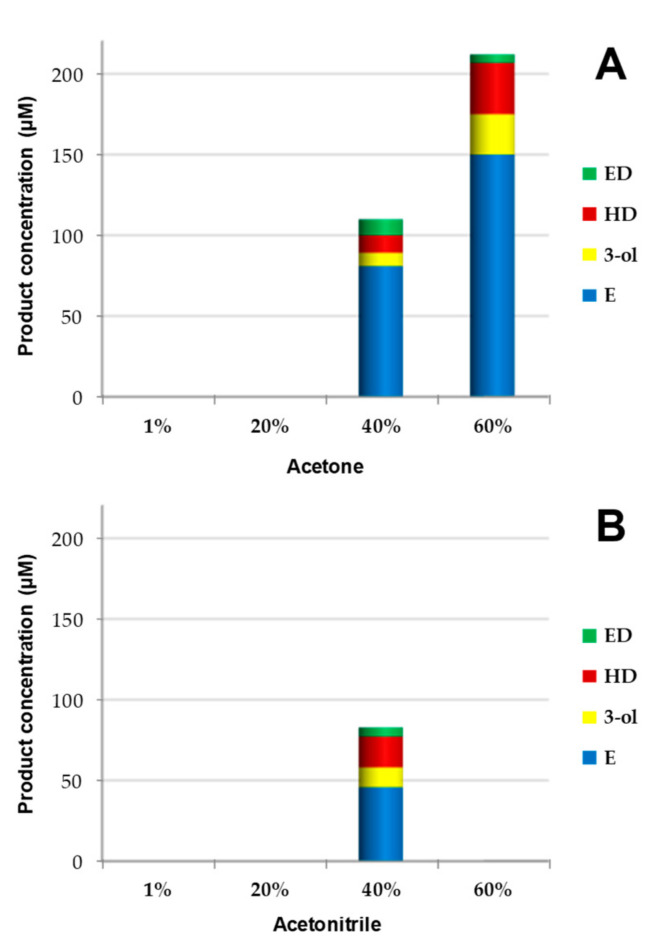
Reactions of *Cgl*UPO (2 µM) with 0.5 mM 1-tetradecene (over 24 h) at several cosolvent concentrations: (**A**) acetone (CH_3_–CO–CH_3_); (**B**) acetonitrile (CH_3_–CN). Identified products: 1,2-epoxytetradecane (E, blue), tetradecen-3-ol (3-ol, yellow), other alkene monohydroxylated derivatives (HD, red), and epoxide monohydroxylated derivatives (ED, green).

The reaction of 1-tetradecene (C_14:1_, 1 mM) with *Cgl*UPO (2 µM) was studied (over 24 h reaction time) under various conditions. Due to the low solubility of alkenes in aqueous media, the cosolvent was the first parameter considered with the testing of different proportions of two solvents commonly used in UPO-catalyzed reactions, i.e., acetone and acetonitrile [31]. Substantial substrate conversion under the formation of different oxygenated derivatives was only observed when the proportion of the cosolvent was kept between 40% and 60% acetone (Figure 1A) or 40% acetonitrile (Figure 1B), with acetone attaining higher conversions, particularly at 60% concentration, in which the products’ concentration was almost twice as high (212 µM vs. 110 µM) than at 40%. When higher acetone concentration (80%) was used in the reaction, no substrate conversion was observed.

**Scheme 1:**
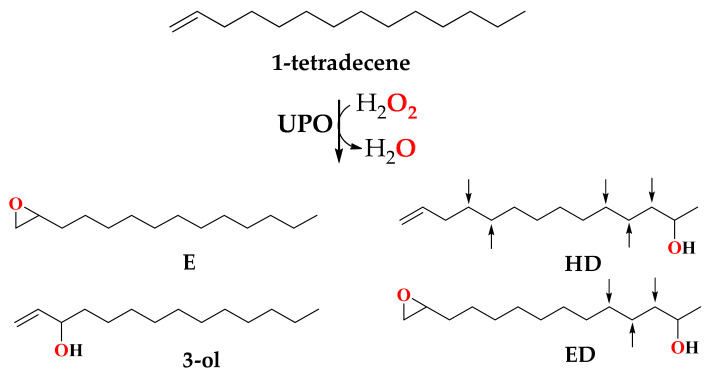
Main products in UPO reactions with 1-tetradecene as substrate including: 1,2-epoxytetradecane (E); monohydroxylated derivatives (HD); tetradecen-3-ol (3-ol); monohydroxylated epoxy-derivatives (ED). Arrows in the formulae of HD and ED indicate alternative positions of the hydroxyl group.

With both solvents, the main derivative of 1-tetradecene was the corresponding epoxide (E; 1,2-epoxytetradecane), although other hydroxylated derivatives (i) at the allylic position (3-ol, tetradecen-3-ol) and (ii) at other positions of the alkyl chain of tetradecene (HD) or of its epoxide (ED) were also produced (Scheme 1, Figure 1). The 1,2-epoxytetradecane (E) was identified by GC-MS analysis using its mass spectrum (Figure S1A) in comparison with those of the NIST library and an authentic standard. The positions of the hydroxyl group in the products (ED, 3-ol and HD) could be deduced from the mass spectra of the silylated derivatives, as illustrated in Figures S1B,C and S2.

Since acetone is not oxidized by the enzyme, increases the solubility of alkenes in water mixtures, and hardly affects the activity of the enzyme [31], it was the solvent of choice for further studies.

The next reaction parameter studied was the cosubstrate type (peroxide acting as electron acceptor and source of oxygen) and its concentration, which is known to be one of the key factors determining UPO performance. On the one hand, high peroxide concentrations are required to achieve adequate yields; on the other hand, too-high concentrations can cause irreversible enzyme deactivation via UPO-compound III and subsequent hydroxyl radical formation [55]. Both hydrogen peroxide (H_2_O_2_) and the milder (and more soluble in organic solvents) *tert*-butyl hydroperoxide (*t*BuOOH) were tested at different concentrations (0.5–5 mM) in the presence of different amounts of *Cgl*UPO (Table 1).

For the same *Cgl*UPO dose (0.5, 1, or 2 µM), a higher product amount was always achieved with 1 mM of H_2_O_2_ (51, 49, and 195 µM, respectively). When *t*BuOOH was used as the cosubstrate, the amount of product was lower in all cases compared to H_2_O_2_ (91, 94, and 28 μM vs. 100, 195, and 134 μM, respectively). Regarding the reaction products (Scheme 1), 1,2-epoxytetradecane (E) was the main product in all cases, together with lower amounts of tetradecen-3-ol (3-ol), other monohydroxylated derivatives (HD) of tetradecene, and several monohydroxylated derivatives of 1,2-epoxytetradecane (ED). There was no clear relationship between the peroxide doses and the relative abundance of the above products (Table 1).

### 3.2. Selective Epoxidation of 1-Tetradecene by Several UPOs

The optimized reaction conditions found with *Cgl*UPO (60% acetone and 1 mM H_2_O_2_) were used to evaluate the selectivity of the different UPOs mentioned above with 1-tetradecene as the substrate. Two pHs (5.5 and 7.0) were tested based on the pH optima of previously reported UPOs [28,40,47]. The results revealed that all UPOs tested were able to convert 1-tetradecene, with r*Cci*UPO achieving the highest levels, followed by r*Hin*UPO and *Cgl*UPO (Table 2).

The pH of the reaction mixture had a significant effect on substrate conversion and all enzymes attained a higher percentage conversion of 1-tetradecene at pH 5.5 than at pH 7.0, with the exception of *Cgl*UPO and *Aae*UPO. In agreement with these results, previous studies reported that a neutral pH was preferred for *Aae*UPO [28] and C*gl*UPO [40] in diverse oxyfunctionalizations, while pH 5.5 was optimal for *Mro*UPO [47]. However, the relative abundance of the differently oxygenated products was not affected by the pH.

Among the UPOs tested, the most selective enzyme regarding epoxidation was *Mro*UPO, producing 96% of 1,2-epoxytetradecane and 4% of the alkene hydroxylated at the allylic position (tetradecen-3-ol, Table 2). The other UPOs were less selective, since, in addition to the epoxide, they generated tetradecen-3-ol together with other monohydroxylated alkene derivatives, substituted either at the subterminal positions ω-1 and ω-2 (*Aae*UPO, r*Cci*UPO, and *Cgl*UPO) or at medium positions (between ω-3 and ω-11) in the case of *Cgl*UPO, r*Hin*UPO, and r*Dca*UPO. Previous studies using 1-tetradecene as the substrate resulted in only 1% conversion with r*Cci*UPO and no reaction with *Aae*UPO, while over 80% and 10% conversion, respectively, were achieved using 7-tetradecene as the substrate under otherwise identical conditions [32].

### 3.3. Selective Epoxidation of Different Long-Chain Alkenes by UPOs

Once the selectivity for 1-tetradecene epoxidation by the various UPOs was verified, a comparative study was performed with a series of long-chain terminal alkenes (C_12:1_–C_20:1_) to determine whether the chain length of the substrates affected their conversion and reaction selectivity.

**Figure 2:**
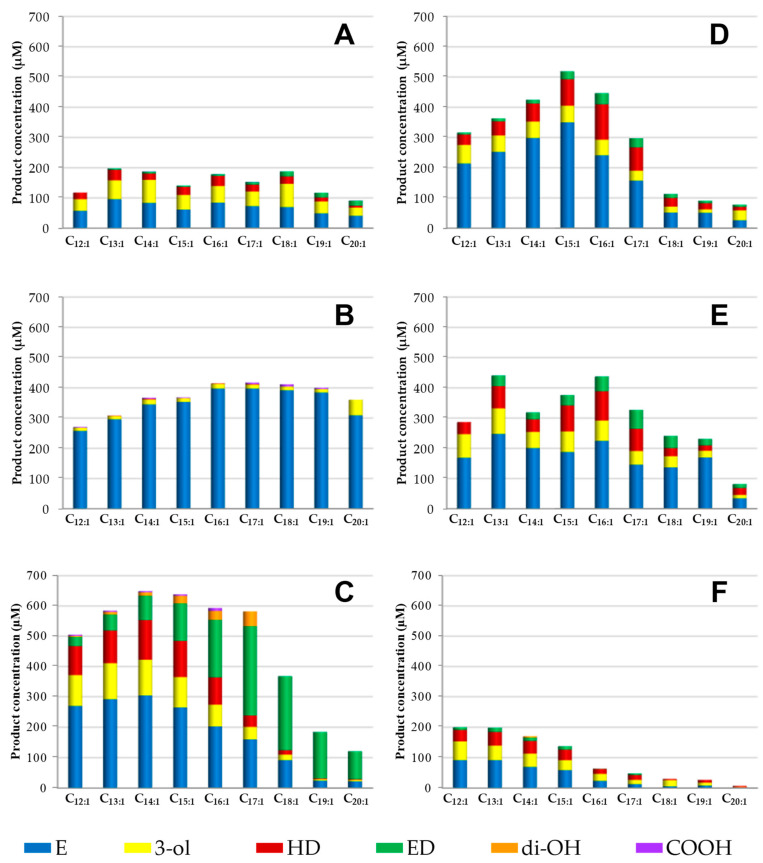
Enzymatic reactions of 1 mM long-chain terminal alkenes (C_12:1_ to C_20:1_) with 3 µM of UPOs. *Aae*UPO (**A**); *Mro*UPO (**B**); r*Cci*UPO (**C**); *Cgl*UPO (**D**); r*Hin*UPO (**E**); r*Dca*UPO (**F**) within 2 h reaction and with 3 mM H_2_O_2_ (in 60% acetone). Products: 1,2-epoxy-alkanes (E, blue), 3-hydroxy-alkenes (3-ol, yellow), monohydroxylated alkenes (HD, red), monohydroxylated epoxy-alkanes (ED, green), dihydroxy alkenes (di-OH, orange), and carboxylic acids (COOH, purple).

GC-MS analyses (as shown in Figure S3 for the 1-tetradecene reactions) revealed that all UPOs transformed the nine alkenes tested, although to different extents (Figure 2 and Table S1). GC-MS analyses of the corresponding controls, with H_2_O_2_ and without enzymes (as shown in Figure S4 for 1-tetradecene reactions), verified that no oxygenation was produced in the absence of enzymes. In general, r*Cci*UPO (Figure 2C) was the most efficient enzyme, transforming up to 650 µM of the substrate (C_14:1_), followed by *Cgl*UPO (Figure 2D), and then by r*Hin*UPO (Figure 2E) and *Mro*UPO (Figure 2B). *Aae*UPO (Figure 2A) and r*Dca*UPO (Figure 2F) were less efficient under the conditions used, only achieving 200 µM products. The chain-length of alkenes had no notable influence on the conversion (at least below C_17:1_), except in the case of *Cgl*UPO and r*Dca*UPO. The alkenes with higher chain lengths (C_18:1_–C_20:1_) were less converted, especially C_20:1_, with the noteworthy exception of *Mro*UPO, which was the only enzyme clearly not affected by chain length.

Among the UPOs tested, the most selective enzyme toward the epoxidation of the different alkenes was *Mro*UPO, producing 96% of 1,2-epoxytetradecane and only 4% of hydroxylated alkene derivatives, the main hydroxy-alkene being tetradecen-3-ol. The other UPOs were not as selective as *Mro*UPO (with *Aae*UPO and r*Dca*UPO being less selective), because, in addition to the epoxide, they generated considerable amounts of other hydroxylated alkene derivatives (mainly at the allylic position, 3-ol). Furthermore, in some cases, the overoxygenation of these products led to the formation of dihydroxylated products and carboxylic acids, especially evident in the r*Cci*UPO reactions (Figure 2 and Figure S3). No ring opening was observed in any reaction.

Regarding the hydroxylated products obtained, the allylic position was favored in all cases, yielding the corresponding alken-3-ol (Figure S2A). *Aae*UPO and r*Cci*UPO were also able to hydroxylate the subterminal positions, yielding the ω-1 and ω-2 hydroxylated alkene derivatives, as shown by mass spectra (Figure S2B,C, respectively). Interestingly, only the UPOs of ascomycetous species (namely *Cgl*UPO, r*Hin*UPO, and r*Dca*UPO) were able to hydroxylate the molecule at different positions of the alkyl chain, resulting in a variety of hydroxylated alkenes (Figure 2D–F). Among the UPOs tested, *Mro*UPO was the most regioselective enzyme, mainly yielding the epoxides as products (Figure 2B and Figure S2), while r*Cci*UPO was the least selective enzyme, always yielding mixtures of four or more products (Figure 2C and Figure S2).

To the best of our knowledge, the epoxidation of long-chain terminal alkenes by enzymes is reported here for the first time. A previous study (only including *Aae*UPO) was carried out with short-chain terminal alkenes (C_3:1_ to C_8:1_) but the product amounts were one to two orders lower (10 to 194 µM) [33]. Variants of P450 BM-3 were reported to epoxidize short terminal alkenes (C_5:1_ to C_8:1_) but not alkenes with more carbon atoms [22]. Likewise, studies with methane monooxygenase showed that this enzyme is unable to oxygenate terminal alkenes with more than five carbon atoms [20].

## 4. Conclusions

The enzymatic epoxidation of terminal alkenes by UPOs is limited, probably due to alkylation of the heme group (or of catalytically relevant amino acid residues) by the epoxidized products, causing inactivation of the enzyme. Despite this, the present results showed how several wild and recombinant UPOs are able to epoxidize long-chain alkenes (from C_12:1_ to C_20:1_) using 60% acetone as a cosolvent.

In these reactions, *Mro*UPO appears to be the most selective UPO, being able to epoxidize the terminal double bond with the corresponding 1,2-epoxyalkane representing over 95% of the reaction products. r*Cci*UPO, although less specific, produced the highest amount and variety of products, such as epoxy-, hydroxy-, and hydroxy-epoxy derivatives; dihydroxy alkenes; and carboxylic acids.

Regarding alkene hydroxylation, the allylic position was preferred by all UPOs. Furthermore, the formation of hydroxylated derivatives at (sub)terminal positions was observed in the reactions with the basidiomycetous UPOs, while additional positions were hydroxylated by the UPOs from Ascomycota.

This work shows for the first time the ability of some UPOs to oxygenate long-chain (C_12:1_–C_20:1_) terminal alkenes, yielding reactive epoxides that are of interest as building blocks in the pharmaceutical, flavoring, and polymer sectors.

## Figures and Tables

**Table 1:** Total (µM) and relative abundance (%) of products (E, 3-ol, HD, and ED, see Scheme 1) from the reactions (24 h, 60% acetone) of 1-tetradecene (1 mM) with several doses of enzyme (*Cgl*UPO) and cosubstrates (H_2_O_2_ or *t*BuOOH).

Enzyme (µM)	Cosubstrate (Type)	Cosubstrate (mM)	Total (µM)	Products (%)			
				E	3-ol	HD	ED
0.5	H_2_O_2_	0.5	11	83	17	0	0
0.5	H_2_O_2_	1	51	64	13	24	0
1	H_2_O_2_	1	49	68	9	13	10
1	H_2_O_2_	5	46	63	10	23	4
2	H_2_O_2_	0.5	100	71	12	14	4
2	H_2_O_2_	1	195	72	12	14	2
2	H_2_O_2_	5	134	73	12	14	2
2	*t*BuOOH	0.5	91	69	11	15	5
2	*t*BuOOH	1	94	68	12	17	3
2	*t*BuOOH	5	28	82	18	0	0

**Table 2:** Total (µM) and relative abundance (%) of products (E, 3-ol, HD, and ED, see Scheme 1) from the reactions (24 h, 60% acetone, 1 mM H_2_O_2_) of 1-tetradecene (1 mM) with several UPOs (2 µM) at pH 7.0 or 5.5.

Enzyme (µM)	pH	Total (µM)	Products (%)			
			E	3-ol	HD	ED
*Aae*UPO	7.0	127	60	21	16	3
	5.5	110	61	21	18	0
*Mro*UPO	7.0	89	95	5	-	-
	5.5	137	96	4	-	-
r*Cci*UPO	7.0	265	43	19	26	12
	5.5	317	44	18	27	11
*Cgl*UPO	7.0	212	71	12	15	2
	5.5	164	70	11	14	5
r*Hin*UPO	7.0	210	58	17	22	3
	5.5	271	58	16	23	3
r*Dca*UPO	7.0	160	44	22	28	4
	5.5	180	45	20	27	8

## Data Availability

Data are contained within the article.

## Supplementary Materials

The following supporting information can be downloaded at: https://www.mdpi.com/article/10.3390/antiox11030522/s1, Figure S1: Mass spectra of epoxy- and hydroxy-epoxy- alkanes, the latter as trimethylsilyl derivatives, from reactions of 1-tetradecene with r*Cci*UPO. (A) 1,2-epoxytetradecane, (B) 1,2-epoxytetradec-13-ol; and (C) 1,2-epoxytetradec-12-ol; Figure S2: Mass spectra of hydroxy-alkenes, as trimethylsilyl derivatives, from reactions of 1-tetradecene with r*Cci*UPO; (A) tetradecen-3-ol, (B) tetradecen-13-ol, and (C) tetradecen-12-ol; Figure S3: GC-MS analysis of 1-tetradecene (1 mM) reactions (2 h) with 3 μM *Aae*UPO (A), *Mro*UPO (B), r*Cci*UPO (C), *Cgl*UPO (D), r*Hin*UPO (E) and r*Dca*UPO (F). The main products are 1,2-epoxytetradecane (E) and several monohydroxy alkenes (1-ol, 3-ol, 12-ol and 13-ol), together with some epoxy derivatives (ED) and dihydroxy (di-OH) and carboxylic (COOH) alkene derivatives. The chromatograms are normalized to same total-ion vertical scale for comparison; Figure S4: GC-MS analysis of 1-tetradecene (1 mM) reactions (2 h) including control reaction with 3 mM H_2_O_2_ and without enzyme (A), enzymatic reaction with 3 μM *Mro*UPO and 3 mM H_2_O_2_ (B) and enzymatic reaction with 3 μM r*Cci*UPO and 3 mM H_2_O_2_ (C); Table S1: Inventory of products in the reactions (2 h, 60% acetone, and 3 mM H_2_O_2_) of nine terminal alkenes (1 mM C_12:1_–C_20:1_) with six UPOs (3 μM) yielding: 1,2-epoxy- (E), 3-hydroxy-(3-ol), other hydroxy- (HD), hydroxy-epoxy- (ED), dihydroxy- (di-OH), and carboxylic-(COOH) derivatives.
