# Supplementary material for: Enzymatic Epoxidation of Long-Chain Terminal Alkenes by Fungal Peroxygenases
Source: Antioxidants (Basel). 2022 Mar 8;11(3):522. doi: 10.3390/antiox11030522 (PMC8944640; doi:10.3390/antiox11030522)
Supplement: Supplementary file 1 [file antioxidants-11-00522-s001.zip › antioxidants-1613015-supplementary.pdf]

SUPPLEMENTARY MATERIALS

Enzymatic epoxidation of long-chain terminal alkenes by fungal peroxygenases

Esteban D. Babot [1], Carmen Aranda [2], Jan Kiebist [3], Katrin Scheibner [3], René Ullrich [4], Martin Hofrichter [4], Angel T. Martínez [5] and Ana Gutiérrez [1,*]

[1] Instituto de Recursos Naturales y Agrobiología de Sevilla, CSIC, E-41012 Seville, Spain. edbabot@irnase.csic.es (E.D.B.); anagu@irnase.csic.es (A.G.)
[2] Johnson Matthey, Cambridge Science Park U260, Cambridge CB4 0FP, UK. Carmen.Aranda@matthey.com (C.A.)
[3] Institute of Biotechnology, Brandenburg University of Technology Cottbus-Senftenberg, 01968 Senftenberg, Germany. jan.kiebist@b-tu.de (J.K.); katrin.scheibner@b-tu.de (K.S)
[4] Unit of Bio- and Environmental Sciences, TU Dresden, International Institute Zittau, 02763 Zittau, Germany. ullrich@tu-dresden.de (R.U.); martin.hofrichter@tu-dresden.de (M.H.)
[5] Centro de Investigaciones Biológicas "Margarita Salas", CSIC, E-28040 Madrid, Spain. ATMartinez@cib.csic.es (A.T.M.)
* Correspondence: anagu@irnase.csic.es; +34 954624711.

This Supplementary Materials includes: Mass spectra of epoxy- and hydroxy-epoxy-alkanes (**Figure S1**) and hydroxy-alkenes (**Figure S2**), the two latter as trimethylsilyl derivatives, from reactions of 1-tetradecene with r*Cci*UPO; Inventory of products in the reactions of nine terminal alkenes with six UPOs (**Table S1**); and GC-MS analysis of 1-tetradecene reactions with several UPOs (**Figure S3**).

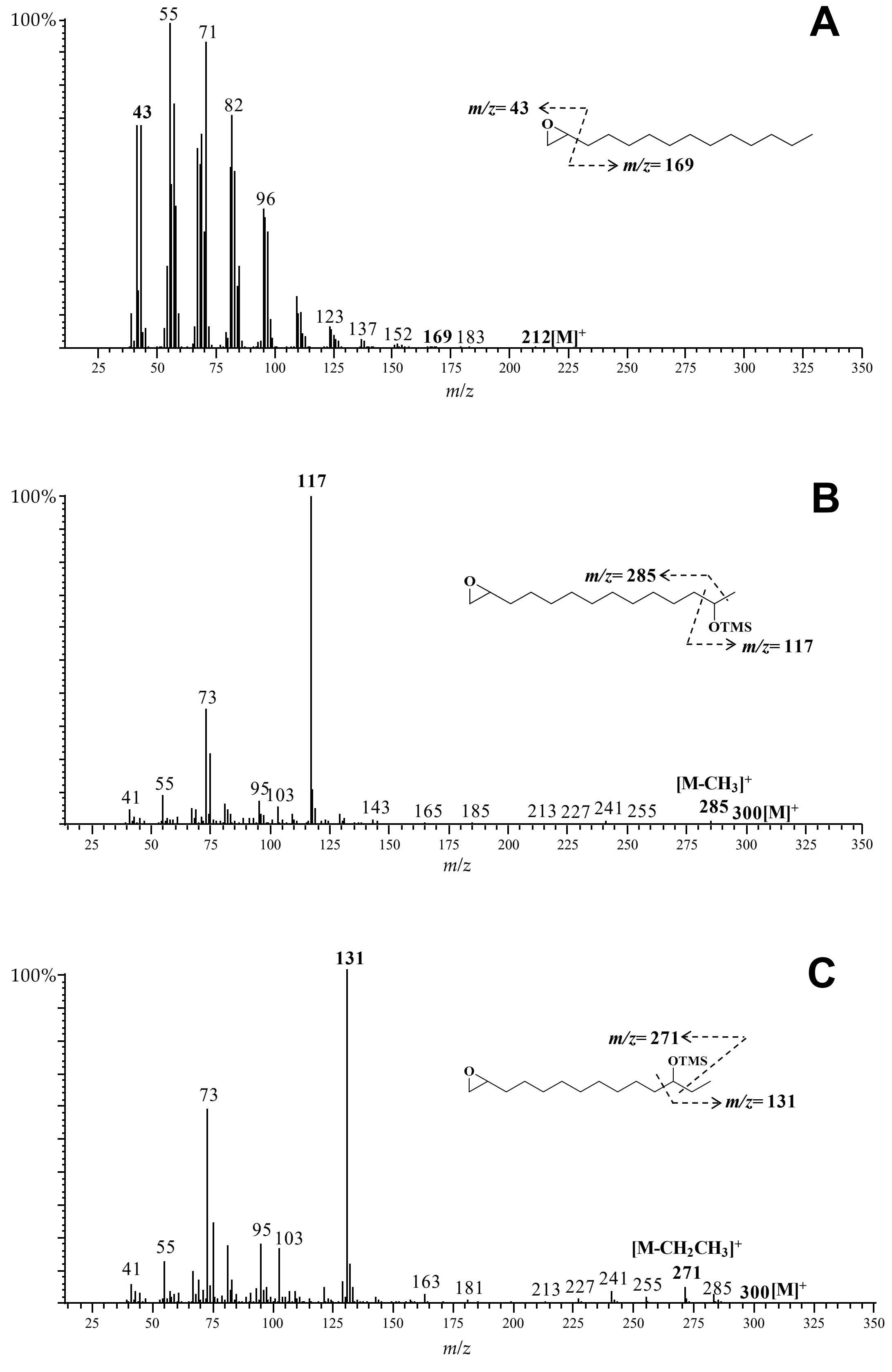

Figure S1. Mass spectra of epoxy- and hydroxy-epoxy- alkanes, the latter as trimethylsilyl derivatives, from reactions of 1-tetradecene with r*Cci*UPO. **A**) 1,2-epoxytetradecane, **B**) 1,2-epoxytetradec-13-ol; and **C**) 1,2-epoxytetradec-12-ol.

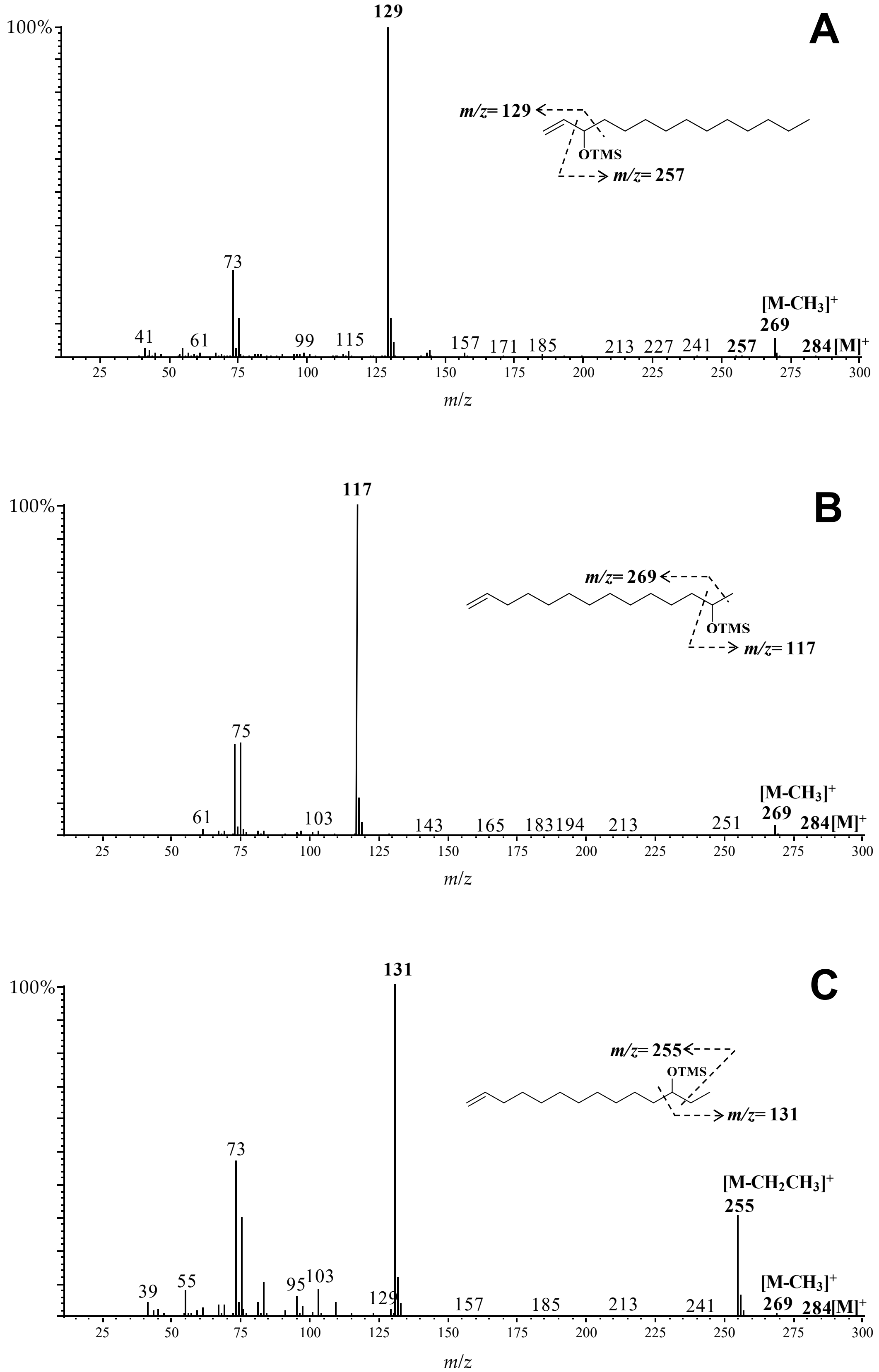

Figure S2. Mass spectra of hydroxy-alkenes, as trimethylsilyl derivatives, from reactions of 1-tetradecene with r*Cci*UPO; **A**) tetradecen-3-ol, **B**) tetradecen-13-ol, and **C**) tetradecen-12-ol.

Table S1. Inventory of products in the reactions (2 h, 60% acetone, and 3 mM H_2O_2) of nine terminal alkenes (1 mM $C_{12:1}$-$C_{20:1}$) with six UPOs (3 μM) yielding: 1,2-epoxy- (**E**), 3-hydroxy- (**3-ol**), other hydroxy- (**HD**), hydroxy-epoxy- (**ED**), dihydroxy- (**di-OH**), and carboxylic- (**COOH**) derivatives.

	**Products** (μM)						
**UPO/alkene**	**E**	**3-ol**	**HD**	**ED**	**di-OH**	**COOH**	**Total**
Aae**UPO**							
1-dodecene	57.5	38.5	22.2	--	--	--	118.2
1-tridecene	96.1	61.8	35.3	4.7	--	--	197.9
1-tetradecene	83.8	75.8	21.8	5.6	--	0.6	187.5
1-pentadecene	61.9	48.1	26.8	4.0	--	--	140.8
1-hexadecene	84.7	54.7	33.7	6.0	--	--	179.2
1-heptadecene	72.7	48.6	22.9	8.4	--	0.9	153.6
1-octadecene	69.6	77.2	24.3	17.0	--	--	188.1
1-nonadecene	49.4	39.3	13.0	15.8	--	--	117.5
1-eicosene	40.8	27.3	6.7	16.8	--	--	91.6
Mro**UPO**							
1-dodecene	257.4	11.0	--	--	--	2.0	270.4
1-tridecene	296.0	10.6	1.3	--	--	--	307.9
1-tetradecene	345.3	15.7	2.7	--	--	3.8	367.4
1-pentadecene	353.4	12.1	--	--	--	2.4	367.9
1-hexadecene	397.5	15.1	2.0	--	--	--	414.6
1-heptadecene	397.5	13.1	1.2	--	--	5.6	417.4
1-octadecene	391.9	12.1	--	--	--	7.2	411.2
1-nonadecene	384.3	11.6	--	--	--	4.0	399.9
1-eicosene	308.8	51.5	--	--	--	--	360.3
**r*****Cci*****UPO**							
1-dodecene	269.6	101.6	96.0	29.8	3.6	4.1	504.7
1-tridecene	291.8	118.5	108.7	51.8	9.5	4.2	584.6
1-tetradecene	304.2	117.4	131.8	80.0	11.1	4.5	649.0
1-pentadecene	264.8	99.6	119.7	123.9	25.4	4.6	638.0
1-hexadecene	202.3	71.3	90.5	189.6	30.0	9.8	593.4
1-heptadecene	159.9	41.3	37.0	294.6	49.1	--	581.8
1-octadecene	91.7	18.3	14.6	243.6	--	--	368.2
1-nonadecene	25.0	4.5	2.6	152.7	--	--	184.8
1-eicosene	22.4	5.1	1.9	92.4	--	--	121.8
Cgl**UPO**							
1-dodecene	214.3	61.5	33.8	7.0	--	--	316.6
1-tridecene	252.3	54.2	46.5	9.9	--	--	362.9
1-tetradecene	298.2	53.8	60.3	11.8	--	--	424.1
1-pentadecene	350.0	54.5	87.7	26.7	--	--	518.9
1-hexadecene	241.1	51.1	117.2	36.9	--	--	446.4
1-heptadecene	157.3	33.2	76.6	30.0	--	--	297.2
1-octadecene	51.6	19.8	28.4	13.6	--	--	113.4

1-nonadecene	50.9	11.5	20.8	7.8	--	--	91.1
1-eicosene	26.6	32.8	10.9	7.7	--	--	78.1
**r*Hin*UPO**							
1-dodecene	168.7	78.2	40.0	--	--	--	286.8
1-tridecene	247.3	85.0	73.5	35.2	--	--	441.0
1-tetradecene	200.5	53.4	41.9	23.3	--	--	319.1
1-pentadecene	187.5	68.2	86.4	33.8	--	--	375.9
1-hexadecene	224.3	67.2	96.5	49.7	--	--	437.7
1-heptadecene	146.3	44.9	73.4	62.2	--	--	326.7
1-octadecene	137.1	36.7	27.3	39.7	--	--	240.8
1-nonadecene	169.4	22.8	17.5	21.3	--	--	231.0
1-eicosene	34.9	11.0	22.7	13.3	--	--	81.9
**r*Dca*UPO**							
1-dodecene	92.3	60.8	37.6	9.5	--	--	200.2
1-tridecene	91.7	47.6	45.0	13.7	--	--	198.0
1-tetradecene	70.1	43.3	41.6	10.8	4.5	--	170.3
1-pentadecene	58.9	33.4	34.2	11.1	--	--	137.6
1-hexadecene	24.3	22.9	15.7	0.8	--	--	63.7
1-heptadecene	13.1	14.0	16.7	4.5	--	--	48.4
1-octadecene	5.7	21.0	3.9	--	--	--	30.7
1-nonadecene	8.0	9.9	9.5	--	--	--	27.4
1-eicosene	1.0	2.7	4.0	--	--	--	7.7

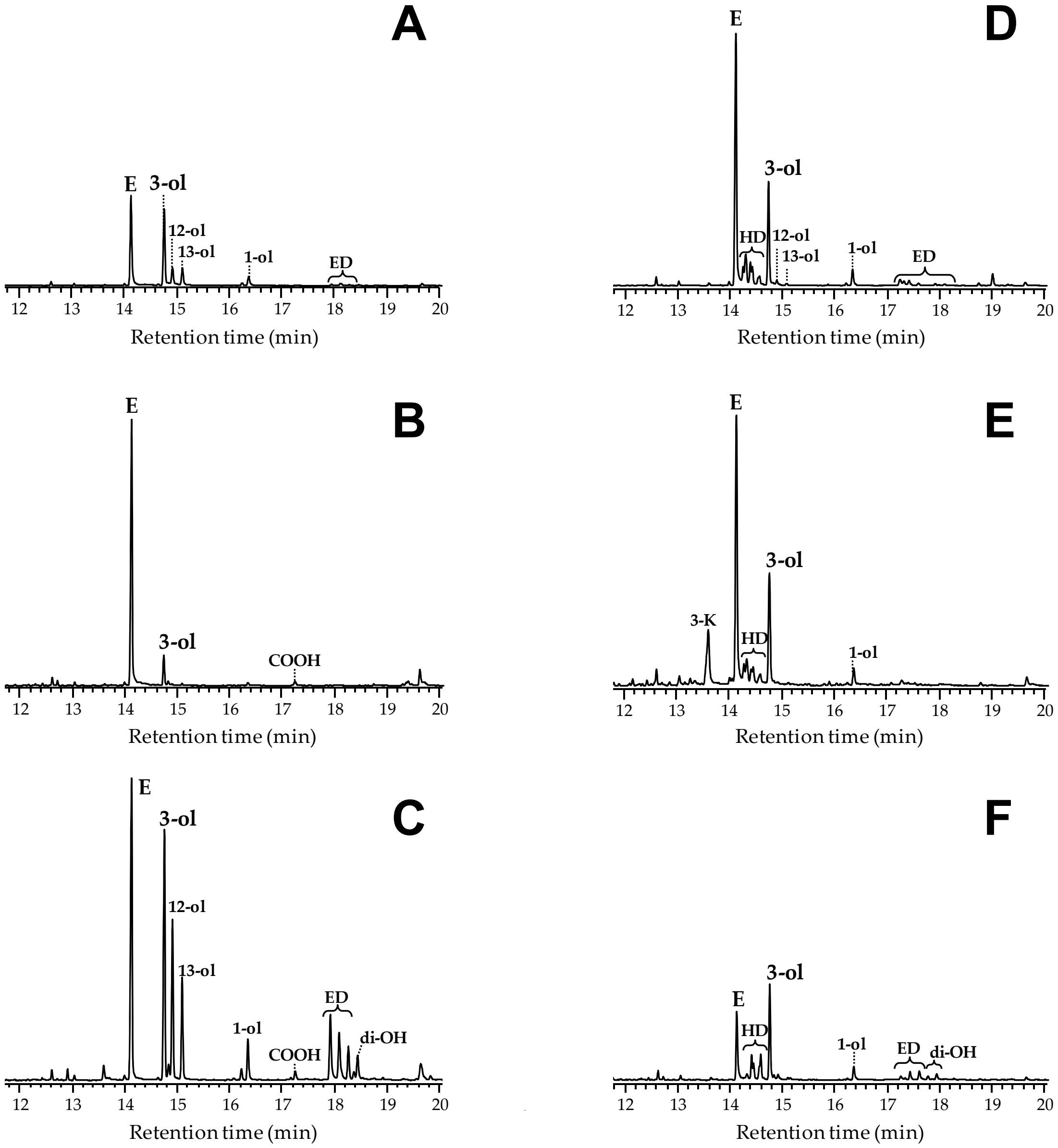

Figure S3. GC-MS analysis of 1-tetradecene (1 mM) reactions (2 h) with 3 µM *Aae*UPO (**A**), *Mro*UPO (**B**), r*Cci*UPO (**C**), *Cgl*UPO (**D**), r*Hin*UPO (**E**) and r*Dca*UPO (**F**). The main products are 1,2-epoxytetradecane (**E**) and several monohydroxy alkenes (1-ol, 3-ol, 12-ol and 13-ol), together with some epoxy derivatives (**ED**) and dihydroxy (di-OH) and carboxylic (COOH) alkene derivatives. The chromatograms are normalized to same total-ion vertical scale for comparison.

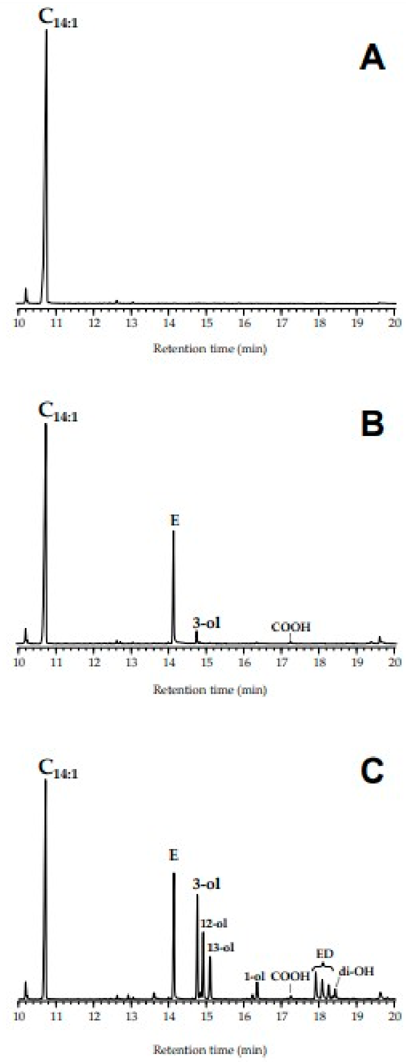

Figure S4. GC-MS analysis of 1-tetradecene (1 mM) reactions (2 h) including control reaction with 3 mM H2O2 and without enzyme (A), enzymatic reaction with 3 µM MroUPO and 3 mM H2O2 (B) and enzymatic reaction with 3 µM rCciUPO and 3 mM H2O2 (C)..
